# Supplementary material for: Change in Caffeine Consumption after Pandemic (CCAP-Study) among University Students: A Cross-Sectional Study from Italy
Source: Nutrients. 2024 Apr 11;16(8):1131. doi: 10.3390/nu16081131 (PMC11053568; doi:10.3390/nu16081131)
Supplement: Supplementary file 1 [file nutrients-16-01131-s001.zip › nutrients-2943428-supplementary.pdf]

**Table S1.** Reasons of energy drinks and energizing supplements consumption.

| Reasons of Energy drinks consumption | n | % | Reasons of supplement consumption | n | % |
|---|---|---|---|---|---|
| To be more concentrated during study | 38 | 31.1 | I need more energy | 49 | 30.4 |
| I like the taste | 28 | 23.0 | I like the taste | 48 | 29.8 |
| I need more energy | 21 | 17.2 | To be more concentrated during study | 32 | 19.9 |
| To stay awake | 18 | 14.8 | To stay awake | 18 | 11.2 |
| To improve my sport performaces | 11 | 9.0 | To improve my sport performaces | 10 | 6.2 |
| Others | 6 | 4.9 | Others | 4 | 5.5 |
